# IVF in Africa: what is it all about?

**Published:** 2019-03

**Authors:** W Ombelet, J Onofre

**Affiliations:** Genk Institute for Fertility Technology, Department of Obstetrics and Gynaecology, Schiepse Bos 6, 3600 Genk, Belgium;; Hasselt University, Department of Physiology, Hasselt, Belgium.

**Keywords:** accessible, affordable, Africa, ART, assisted reproductive technologies, childlessness, developing countries, infertility, IVF

## Abstract

Infertility is a universal problem with the highest prevalence in low-resource countries, particularly in sub-Saharan Africa where infection-related tubal damage is the commonest cause. It is estimated that more than 180 million couples in developing countries suffer from primary or secondary infertility. In most African countries, the social stigma of childlessness still leads to isolation and abandonment. Differences between the developed and developing world are emerging because of the different availability in infertility care and the different socio-cultural value surrounding procreation and childlessness.

Although reproductive health education and prevention of infertility are number one priorities, the need for accessible diagnostic procedures and affordable assisted reproductive technologies (ART) is very high. The success and sustainability of ART in resource-poor settings will depend, to a large extend, on our ability to optimise these techniques in terms of availability, affordability and effectiveness.

Different new innovations and techniques can make the diagnostic work-up and treatment through assisted reproductive technologies (ART), including in-vitro fertilization (IVF), more affordable. These include automated smartphone-based assays for semen analysis and simplified IVF culture systems. The initiative of African Network and Registry for Assisted Reproductive Technology (ANARA) to register all IVF cycles in Africa needs our support and will be of paramount importance in the future.

The hurdles to implement ART in most African countries are numerous and although more and more IVF centres are founded, the accessibility to ART remains very low.

## Introduction

Infertility is a global health issue. It has been estimated that 8 to 12% of the couples worldwide are infertile with 9 % currently cited as the global average. Remarkably, these numbers are similar between more and less developed countries, although the reason for infertility might differ (Rutstein and Iqbal, 2004; Boivin et al., 2007; Ombelet et al., 2008). On the other hand, in some areas of Sub-Saharan Africa it seems that up to 30% of the couples are infertile (Nachtigall, 2006). Inhorn reported a prevalence as high as 32 % in Namibia, as she described areas of central and southern Africa as “the infertility belt” (Inhorn, 2003).

**Figure 1:**
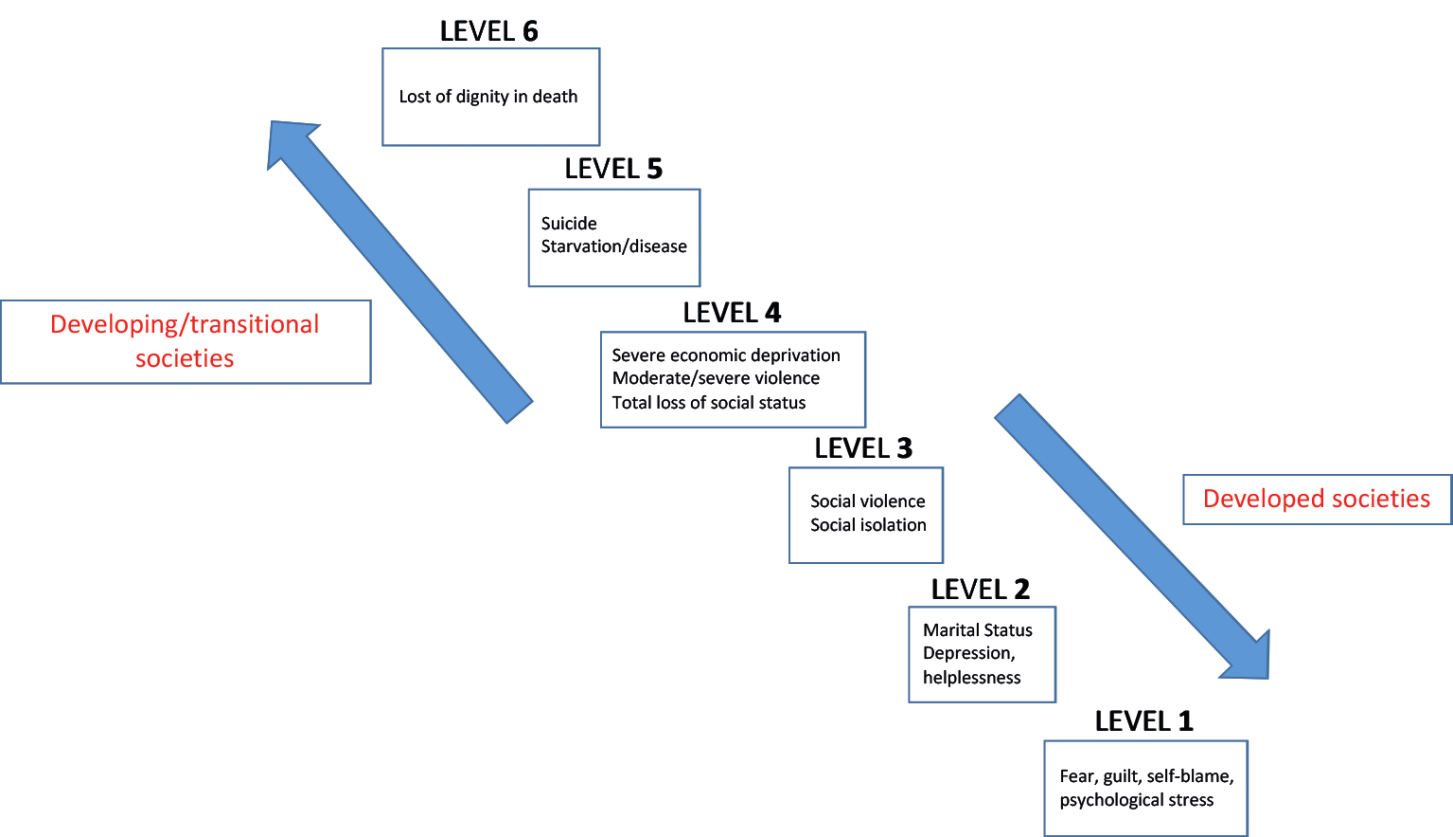
Continuum of the consequences of infertility and childlessness: In developed countries, the consequences of infertility rarely extend beyond level two; in developing countries, at least in Asia and Africa, the consequences of infertility are infrequently as mild as level three (Daar and Merali, 2001).

Compared to Western societies the consequences of involuntary childlessness are usually much more pronounced and creating broader problems in Africa, particularly for women. Childless women are frequently stigmatised, isolated, ostracized, disinherited and neglected by the entire family and even the local community (Daar and Merali, 2002; Dyer et al., 2005; Pilcher, 2006, Van Balen and Bos, 2009) (Figure 1). This may result in physical and psychological violence, polygamy and even suicide. Because many families in developing countries completely depend on children for economic survival, childlessness must be regarded as a social and public health issue and not only as an individual medical problem. According to the results of a systemic analysis by Dyer and Patel (2012) infertility may cause impoverishing health costs as well as economic deprivation secondary to social consequences.

## IVF in Africa: the history

With the exception of Egypt and South Africa, until 2001 there has been little or no interest in subfertility prevention and treatment in Africa. Most scientific papers dealing with the issue of childlessness and the need for infertility care in Africa were published by anthropologists, sociologists and epidemiologists. They described the severe socio-cultural, psychological and economical consequences of being childless in Africa, especially for women (Dyer et al., 2004; 2005; Van Balen et al., 2001; Van Balen and Bos, 2009).

In 2001 the World Health Organization (WHO) organized a meeting in Geneva entitled “Medical; ethical and Social Aspects of Assisted reproduction”. As a result of this expert meeting the following recommendations were given: (1) infertility should be recognized as a public health issue worldwide, including developing countries, (2) policy makers should give attention to the needs of infertile patients, (3) infertility management should be integrated into national reproductive health care programmes and (4) ART has to be complementary to other ethically acceptable socio-cultural solutions to infertility. Almost two decades later, we have to admit that the worldwide attention and policy concerning infertility in Africa has not changed at all.

The next important step towards a better understanding of the infertility problem in low- and middle income countries (LMIC) was the foundation of a “Special Task Force” (STF) dedicated to infertility in developing countries by ESHRE (European Society of Human Reproduction and Embryology) in 2006. Together with the Genk Institute of Fertility technology and The Walking Egg non-profit organization (TWE npo) an expert meeting on the topic of “Developing countries and infertility” was organized in Arusha, Tanzania, in December 2007. Thirty-seven experts from all over the world, including representants of the major scientific societies (FIGO, IFFS, ESHRE,ISMAAR,WHO), sociologists, ethicists, anthropologists, infertility specialists, embryologists, health economists, journalists and a representative of the government of Uganda attended the meeting. It was concluded that the major challenge would be to reduce costs of laboratory procedures namely fertilization and culture of eggs and embryos and also to decrease the costs associated with ovarian stimulation for IVF and/or ICSI. Importantly, according to this group of experts more research on social, cultural, ethical, religious and juridical aspects of infertility in LMIC was urgently needed.

The statement of affordability was confirmed by many publications from African authors, all of them, stressing the importance of access and quality (Ndegwa, 2016; Osei, 2016). The only true hope for most Africans struggling with unintended childlessness lies in the introduction of affordable ART services. The major challenge is to reduce the fixed and running costs of an IVF laboratory, the costs of the diagnostic procedures used in an infertility work-up and the costs associated with ovarian stimulation medication and the equipment set-up. Another important foreseeable barrier to low-cost ART is the unmet need for adequately trained personnel (Ndegwa, 2016).

## An urgent need for accessible and affordable IVF in Africa

**Figure 2:**
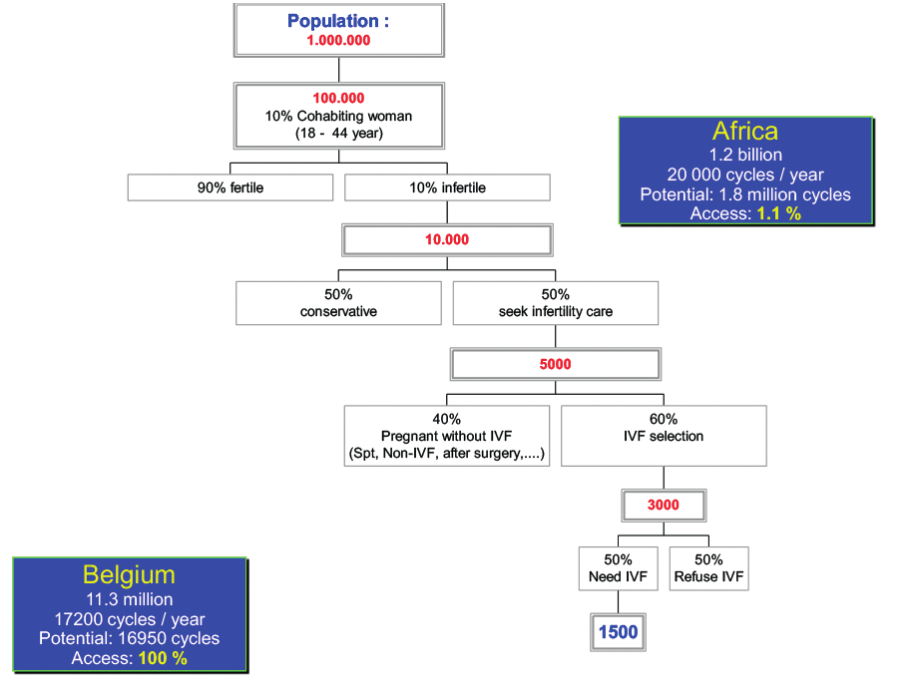
Access to IVF procedures: estimated need of IVF cycles per million (population) (Fauser et al., 2002). Calculation for Belgium (reimbursement of 6 IVF cycles per woman) and Africa. (If 10 % of the population consist of cohabiting women aged 18-44 years, taking into account that 10 % of these have a fertility problem, among them 50 % will seek infertility care. Of the remaining 5000 infertile couples 2500 would qualify for IVF due to persistent infertility and 500 because of tubal factor or severe male infertility. Assuming that only half of them will accept

It was calculated before that there should be 1500 IVF cycles per million people to meet the population demand (Fauser et al., 2002) (Figure 2).

To compare, in Belgium we have a reimbursement policy for six IVF cycles per patient in a lifetime, the financial burden for the patients is minimal. According to the most recent 2016 data, 17191 IVF/ICSI cycles were performed for a population of 11.3 million, this means 1513 attempts per million, exactly what we predicted many years ago when negotiating the reimbursement policy with the government (Ombelet et al., 2005). The African population is actually estimated to be 1.2 billion; therefore 1.8 million IVF cycles should be done to meet its current population demand. Yet, according to the published data, less than 1.5 % of the African population has access to assisted reproduction (Figure 2).

It is difficult to find out how many centres are nowadays performing IVF in Africa, but the number is growing day by day. The African countries with the highest number of IVF centres are Egypt, Ghana, Kenia, Nigeria and South Africa. According to the SARA (South African Registry for Assisted Reproductive Techniques) report 4995 ART cycles were performed in 2014 in 15 IVF centres, this means that even in South Africa only 6.4 % of the need for ART was met.

In the World Report on Assisted Reproductive Technology (Adamson et al., 2017), data from Africa represented only 1% of global ART activity although the African population actually contributes for 16.7 % of the world population.

**Figure 3:**
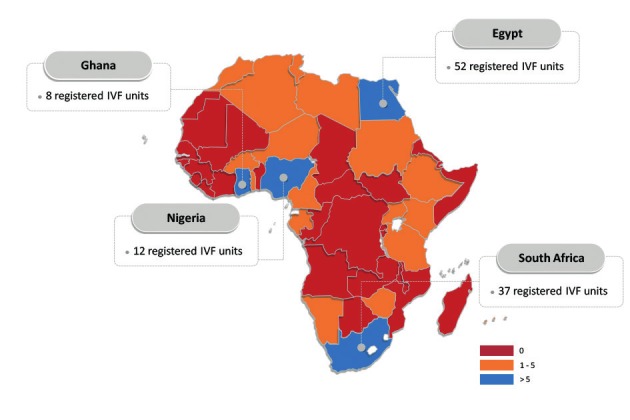
Overview of the number of registred IVF units in the different African countries.

Table I gives an overview of the number of IVF units in the different continents. The differences between the countries are striking. Table IIa, IIb and Figure 3 describe the availability of IVF centres in the different African countries. 

**Table Ia:** — Twenty-one out of 54 African countries have at least one (self-) registered* IVF unit.

Algeria	2	Uganda	4
Burkina Faso	1	Egypt	52
Cameroon	1	Gabon	1
Ethiopia	1	Kenia	3
Ghana	8	Mauritius	1
Libyan Arab Jamahiriya	2	Niger	2
Morocco	2	South Africa	37
Nigeria	12	Tanzania	1
Namibia	1	Tunisia	2
Sudan	2	Zimbabwe	1
Togo	2		

Total of 151 IVF units were (self-)registered in Africa in 2019.

The African network and registry for assisted pro-ductive technologies (ANARA) counts with only 55 registered IVF-units, namely in Egypt, Nigeria and South Africa.

**Table Ib:** — Thirty-three out of 54 African countries (61%) do not have any (self-) registered* IVF unit services.

Angola	Botswana	Comoros
Central African Republic	Chad	Equatorial Guinea
Congo	Djibouti	Guinea
Eritrea	The Gambia	Lesotho
Guinea-Bissau	Kiribati	Malawi
Liberia	Madagascar	Mayotte
Mali	Mauritania	Nauru
Mozambique	Benin	Seychelles
Rwanda	Sao Tome and Principe Senegal	Swaziland
Sierra Leone	Somalia	
Western Sahara	Zambia	
Burundi	Cape Verde	
African countries without an IVF unit: 33		

## IVF: attempts at lowering the costs of IVF

**Figure 4:**
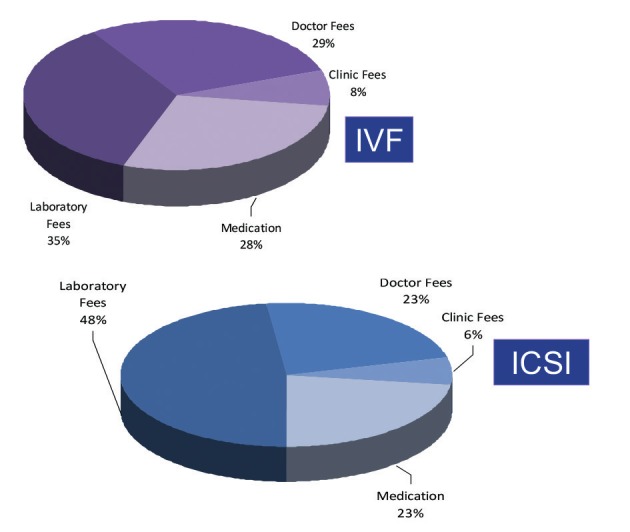
Cost analysis per IVF and ICSI procedure in private centres in South Africa (Huyser and Boyd, 2013).

According to a calculation made by C. Huyser (Pretoria, South Africa) the average percentage of the major cost-drivers of an IVF cycle in South Africa in 2012 were the following: 8% of costs are allocated for clinic fees, 28% to medication, 29% to clinicians’ fees & consultations, and 35% for laboratory fees (for use of equipment and the laboratory, disposables, culture media and staff expenditures). (Huyser and Boyd, 2013). For ICSI the figures were very comparable (Figure 4).

The suggested strategies to reduce the cost of infertility care and infertility treatment are the following:

A simplified “one-stop clinic for the diagnosis of infertility

Standardized investigation of the couple at minimal costs is possible and undoubtedly will enhance the likelihood that infertile couples, both men and woman, will visit infertility centres. A detailed description of how to organize a one-stop diagnostic clinic was described by Ombelet et al. (2012) with the following.

Initially, a questionnaire will be provided to both partners and can be adapted to the local situation in the specific locations and countries. Screening for infections and STDs can be done by using low-cost affordable screening tests. Since tubal obstruction associated with previous pelvic infections is the most important reason for infertility in many African countries, hysterosalpingography and/or hystero-salpingo-contrast-sonography are affordable techniques to detect this problem, easy to perform and without major costs. A standard gynaecological and fertility ultrasound scanning of the uterus and the ovaries can easily be done. Combining these techniques with an accurate medical history will identify the majority of women’s infertility causes, such as ovulatory disorders, uterine malformations and tubal infertility.

Male factor infertility can be evaluated by a simple semen analysis. Semen analyses can be performed by well-trained paramedical personnel, which is an important advantage for developing countries. Very recently, a few smartphone-based diagnostic assays for semen analysis are described in excellent papers showing a nearly 98 % accuracy when compared to regular time-consuming and more expensive sperm quality assessments, well-known for their lack of standardization and methodological problems. It will be crucial to ensure that these new techniques remain affordable (Kobori et al., 2016; Kanakasabapathy et al., 2017; Agarwal et al., 2018).

Due to the high prevalence of uterine factors (Ashermann disease, intrauterine polyps, myomas, etc) investigating the cavity is crucial. Office mini-hysteroscopy to investigate intrauterine abnormalities has been simplified in its instrumentation and technique, so that it can become a non-expensive diagnostic technique accessible for every gynaecologist, provided that they have followed appropriate training (Ombelet and Campo, 2007; Campo et al., 2014).

All the procedures of the one-day diagnostic clinic can be performed by a small team of health care providers within a short period of time in an inexpensive setting (Ombelet and Campo, 2007). Medical results and conclusions from all the exams must be discussed with the couple on that same day to propose a management strategy.

More studies are needed to assess the reproducibility of ‘one-stop infertility clinics’ in African countries.

Low-cost mild ovarian stimulation protocols for IVF

In order to make infertility care more affordable in Africa, effective, cheap and safe stimulation schemes for IVF need to be established. A review of the literature clearly shows the value and effectiveness of mild ovarian stimulation protocols in ART settings (Verberg et al., 2009). The use of clomiphene citrate (CC) or tamoxiphene, very cheap oral drugs, in combination with low-dose recombinant FSH or hMG (gonadotrophins) has been proven to be an optimal alternative with acceptable results, minimal side effects and a very low complication rate (Verberg et al., 2009; Ferraretti et al., 2015; Nargund et al., 2017). Monitoring of follicular development in an IVF cycle, as well as the timing of the hCG administration can be done solely on sonographic criteria with basic inexpensive ultrasound equipment thereby avoiding the need of expensive endocrine investigations.

Simplified IVF laboratory procedures

Another major challenge is to reduce costs of laboratory procedures, namely fertilization and culture of eggs and embryos for IVF. Different options and approaches have been developed or are presently being field-tested with promising results.

As part of the Walking Egg Project we developed a new simplified method of IVF culturing, called the TWE lab method (Van Blerkom et al., 2014). With this new system, specifically designed for low resource settings, we can avoid the high costs of medical gases, complex incubation equipment and infrastructure typical of IVF laboratories in high resource settings. For insemination of the eggs, we only use 2000 - 10000 motile washed spermatozoa per oocyte. To this date, this technique has delivered very promising results, making it reliable and convinient for an estimated >60 % of the actual IVF/ICSI population.

This technique benefits from improved embryo development conditions. Indeed, by using this technique, embryo development, from insemination to transfer, is undisturbed and within the same tube until embryo transfer, thus, we can avoid many problems frequently occurring in regular IVF laboratories, such as unwanted temperature changes, air quality problems, etc. 

Up to March 2019 a total number of 150 healthy babies have been born after using this technique with an unexpected low prevalence of low-birth weight and prematurity. According to our preliminary results, the perinatal outcome of babies born after using the simplified Walking Egg IVF culture system is very reassuring (Genk data, not published yet).

Intravaginal fertilization and culturing is another simplified method that has been used since many years (Mitri et al., 2015). A tube filled with culture medium containing the oocytes and washed spermatozoa is hermetically closed and placed in the vagina. It is held intravaginally by a diaphragm for incubation for 44 to 50 hours, also with very promising results.

## Hurdles and misunderstandings surrounding the implementation of assisted reproduction in Africa

The biggest obstacle in implementing health policies which consider infertility as a problem in Africa is the widespread belief that infertility is not a pressing problem in poor developing countries. Yet, as abovementioned, in these settings fatal and contagious diseases remain uncontrolled. Because infertility as such is not directly life-threatening it remains an entirely neglected problem in most African countries despite the devastating social, psychological, economical and personal consequences of being childless.

Above this, inadequate or complete lack of rules and regulations concerning treatment conditions and commercial interests may lead to unethical practices in some African centres.

Two arguments are always cited when talking about the issue of “infertility care” in resource-poor countries:

The “limited resources” argument

**Figure 6:**
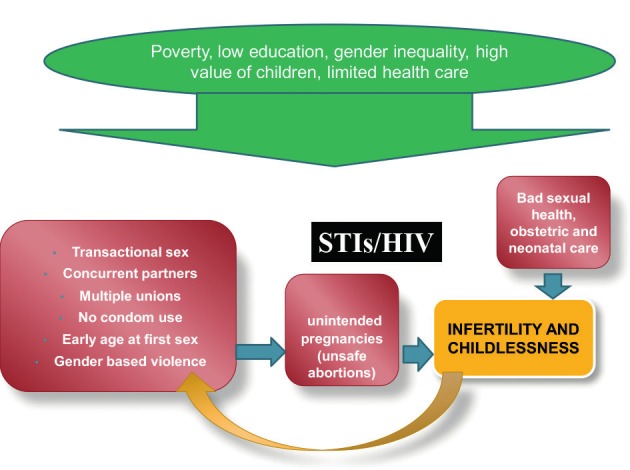
Association between infertility and other socio-cultural determinants and high sexual behavior in Africa.

Can expensive techniques be justified in countries where poverty is still an important issue and where health care systems still struggle with the immense problem of infectious diseases such as malaria, tuberculosis, gonorrhoea and HIV? The strong competition for funding is a reality in most African countries leaving little space for expensive infertility treatment. Most health care providers argue that the limited recourses should only be given to programs focussing on reducing STDs, postpartum and post abortion complications rather than offering high-technology treatments to infertile couples. Prevention and education rather than cure. Nevertheless, it has been proven that HIV is 3 times more prevalent in infertile couples when compared to fertile controls in the same population (Dhont et al., 2011). HIV and infertility share the same determinant of high risk sexual behaviour (Figure 6, Figure 7). Public solutions are being applied for HIV, for infertility the solution is mainly found in the private sector. It is striking that budgets for HIV research are huge and the information on HIV is easily available while the contrary is true for infertility.

The “overpopulation” argument

The argument of overpopulation suggests that in countries where overpopulation poses a demographic problem, infertility management should not be supported by the government.

It is well known that the world population is expected to increase to more than 9 billion in 2050. This increase will be most prominent in the poorest areas such as sub-Saharan Africa and the South East Asia. Therefore, the idea of infertility treatment in Africa often evokes a feeling of discomfort and disbelief. A large part of the world population claim that helping infertile couples in these areas contradicts the interests of the countries and the world at large. However, this narrow approach contradicts human rights in general and, in particular, reproductive rights (Dhont, 2011).

On the other hand, many developing countries already succeeded to drop their global fertility rate (number of children per woman) and the United Nations data clearly show that in the majority of developing countries the mean fertility rate has already dropped from more than 5 to 2.6 and is expected to decline to 1.92 by mid-century, i.e. below the replacement level of 2.1 (Ombelet et al., 2011). This doesn’t mean that family-planning programmes are not important anymore, but implies that infertility care should be part of the concept of family planning, emphasizing that family planning not only refers to prevention of unwanted pregnancies, but also includes promoting the chance of pregnancy in case of involuntary childlessness.

Moreover, the expected population growth in developing countries cannot be attributed to high fertility rates in the first instance anymore. As in most developed countries, an improved life expectancy will be the most important factor considering world population growth. Even in the least developed countries life expectancy is going to rise from an average of 51 years currently to 67 years in 2045-2050 which highlights the important issue of population ageing.

Hence, even if infertility treatment is made accessible in African countries it would probably account for less than 1 % of all deliveries. Increasing efforts on family planning and health education should readily overcome this small contribution to the fertility rate.

## Registration of African ART cycles

The African Network and Registry for Assisted Reproductive Technology (ANARA) was established in 2015 as a research network and platform for communication and information sharing the practice and outcomes of ART in sub-Saharan Africa. This network is modelled after the successful Latin American Network and Registry for Assisted Reproductive Technology (RLA). Importantly, the ANARA counts with its own data collection software, free for participating ART centres. The software collects case-by-case data which is the superior method of ART data collection when compared to retrospective summary data. The ANARA collects data from two settings: (1) directly from participating ART centres in countries which have adopted the ANARA software for national data collection or where no national registry exists and (2) from national ART registries in countries which collect their own national data through a different software program. The registry arm of ANARA collects and disseminates data on the availability, effectiveness and safety of ART in Africa.

Regional and national fertility organizations have expressed their support for ANARA very recently. This includes GIERAF (Groupe Inter Africain d’Etude, de Recherche et d’Application sur la Fertilité) founded in 2009. The GIERAF aims to promote (1) prevention of the avoidable causes of infertility, (2) the training of practitioners in the field of reproduction and (3) exchanges and partnerships with scientific societies concerned with the problems of medicine and the biology of human reproduction. Thirteen countries are involved: Bénin, Burkina Faso, Cameroon , Congo, Ivory Coast, Gabon, Mali, Niger, Democratic Republic of Congo, Senegal and Togo. They presented the 2013 data on 523 oocyte aspirations with a live birth rate per transfer of 26.9 % for IVF and 29.0 % for ICSI (Moîse Fiadjoe, personal communication, 2017).

Dyer et al. (2019) recently reported the 2013 data of ANARA representing centres in 13 African countries. Regional ART utilization could not be established due to large inter-country variations and insufficient data. Deliveries were reported for only 56.1% of pregnancies and the remainder were lost to follow-up. Nevertheless, this first report from the ANARA indicates a willingness of ART centres to voluntarily report and monitor utilization and outcomes of ART, which reflects a rising standard of ART in Africa. Although the future for ART registration in Africa looks bright, one should be aware of the fact that a substantial number of African IVF clinics still don’t share their data. It is anticipated that more centres and countries will join the ANARA to continue this trend.

## Major challenges and hurdles in African settings

Although more than 98 % of the population in Africa can’t afford IVF because it is too expensive or because of a lack of IVF centres, establishing local low cost IVF programs seems to be a low priority, even from local health care providers despite claiming the contrary. Our first experience with the Walking Egg Project in Sub-Saharan Africa showed that considerable opposition from established and expensive IVF centres can be expected against low-resource settings, but above this it has become apparent that despite claims for a huge unmet need for IVF services made by well-intentioned individuals, medical institutions and government officials, they are not really ready or waiting for a viable option to be provided at the moment.

Beside this, many hurdles can be expected when implementing low-resource settings in Africa.

In almost all African countries there is a lack of well trained fertility specialists, embryologists and IVF-nurses. They are mostly trained for a short time in India and elsewhere, but in many cases the expertise is not good enough to ensure enough experience in the field of assisted reproduction (Adageba et al., 2015). African training programmes for the various key personnel in ART centres are lacking and only available in Egypt and South-Africa.

The lack of a reliable supply of good quality drugs, culture media and other important consumables is another serious constraint when running or setting up an IVF centre. In Ghana drug supply is sometimes erratic and most relevant consumables such as egg-retrieval needles, transfer catheters, petri dishes and culture media have to be imported from different countries. Subsequently, delays in delivery are frequent and high import duties contribute significantly to the cost of treatment (Adageba et al., 2015). Also, prices for assisted reproduction related-drugs vary widely between different African countries, often even more expensive than in western countries.

Furthermore, because the companies involved in the setting-up of new IVF-centres are often based in foreign countries, many centres regularly face problems with the maintenance of the equipment due to the cost of bringing-in experienced service technicians/engineers from abroad.

In many African countries, a stable power supply is not available leading to frequent, even daily power cutages and fluctuations although a stable power supply is a necessary requirement in the IVF laboratory. To run a successful IVF programme requires standby generators, batteries and powerful UPS systems to guarantee optimal laboratory conditions (Adageba et al., 2015). This surely contributes to the costs.

Table II summarizes the factors associated with the problem of accessibility to infertility care in Africa.

**Table II:** — Global access to infertility care in Africa: facts, views and vision. (ART = Assisted Reproductive Technologies, STDs = Sexually Transmitted Diseases, OHSS = Ovarian Hyperstimulation Syndrome, NGOs = Non-Governmental Organisations

FACTS	
Prevalence of infertility: similar to Western countries	
Negative consequences of childlessness are much stronger	
Prevention and alternative methods are not always successful	
↑ secondary infertility due to STDs and unsafe abortions / deliveries	
HIV and infertility: ↑ prevalence of HIV in infertile couples	
HIV and infertility: very different in how the issue has been treated by the international community	
Access to IVF in Africa: less than 1.5 %	
**Arguments contra** global access to infertility care	
	Overpopulation
	Limited resources
	Problem of funding: “the battle for money’ between initiatives on reproductive health care
**Arguments pro** global access to infertility care	
	↑ Demand from developing countries
	ART techniques can be simplified
	Social justice and equity
VIEWS	
A need for ↑ reproductive health care education	
A need for ↑ prevention programmes	
Raising awareness: support of media and patients networks needed	
Implementation of more and accessible infertility centres → Urgent need for simplified, safe and effective methods (diagnostic procedures and ART)	
Prevention of complications is crucial: OHSS, multiple pregnancies	
Facilities to handle complications have to be available, including facilities for surgery	
VISION	
Simplified methods of infertility care will be available in the near future	
The demand from Africa to introduce ART will increase	
The implementation of accessible infertility centres should be part of an integrated reproductive care programme including family planning and contraception, mother care and reproductive health.	
Foundations, NGOs and international societies have to be convinced about the value of this project	

## The Walking Egg Project

The Walking Egg non-profit organisation (TWE npo) was founded in 2010 by scientists and an artist. Right from the start The Walking Egg has opted for a multidisciplinary and global approach towards the problem of infertility and in cooperation with the STF on “Developing countries and infertility” of the European Society of Human reproduction and Embryology (ESHRE) and the WHO (Ombelet, 2014). The project aimed to raise awareness surrounding childlessness in resource-poor countries and to make infertility care in all its aspects, including assisted reproductive technologies, available and accessible for a much larger part of the population.

By simplifying the diagnostic and IVF laboratory procedures and by modifying the ovarian stimulation protocols for IVF, assisted reproductive techniques should be offered at affordable prices in the near future.

The Walking Egg also aims to integrate infertility care within the concept of family planning, emphasizing that family planning is not only preventing unwanted pregnancies but also includes promoting the chance of pregnancy in case of involuntary childlessness.

The ultimate goal of The Walking Egg is to work towards a world with less suffering caused by infertility. Figure 7 describes the setting-up of a Walking Egg centre in Accra, Ghana, building the centre, training the team in Genk, batching of treatment cycles and the birth of the first baby on August 7, 2017.

**Figure 7a:**
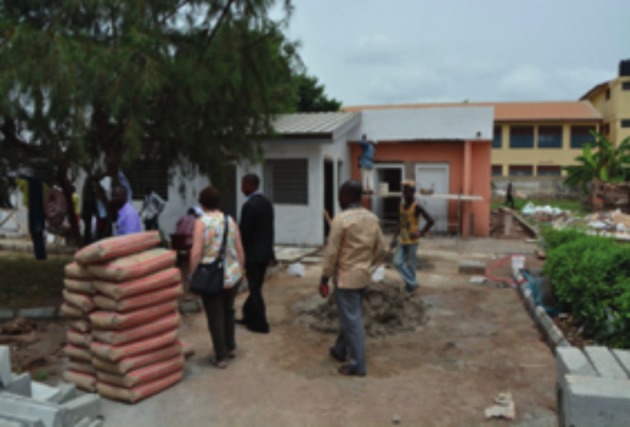
Building the centre with the (financial) support of the Pentecost Church of Ghana.

**Figure 7b:**
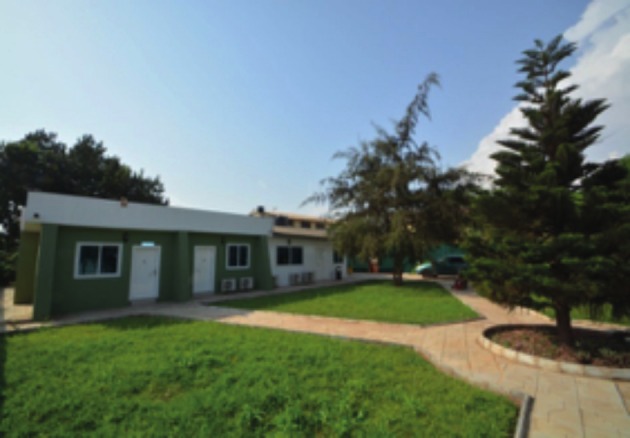
The final result.

**Figure 7c:**
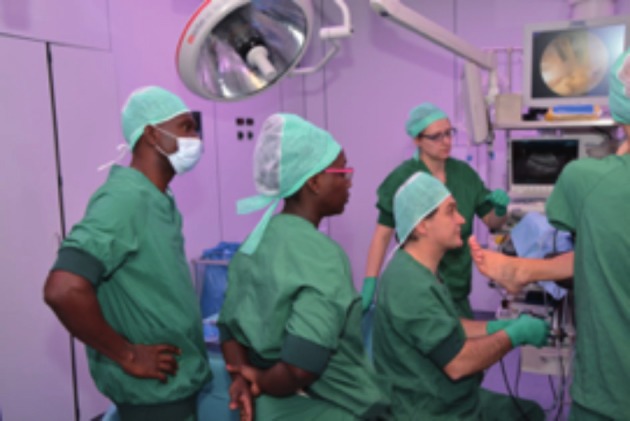
The Accra team in Genk (Belgium): training in hysteroscopy by Dr. Campo.

**Figure 7d:**
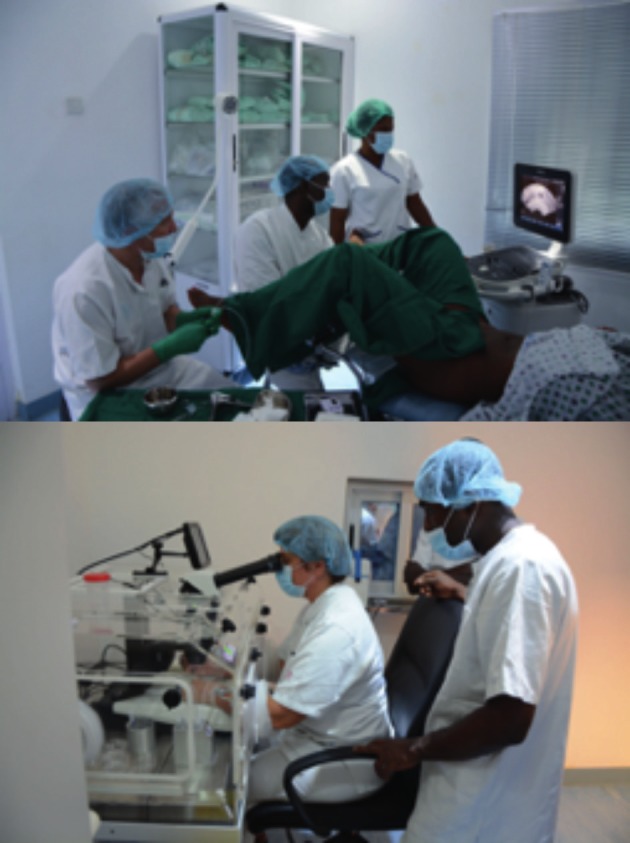
Starting-up the first IVF trials with a batch of selected couples. Oocyte retrieval and laboratory performance.

**Figure 7e:**
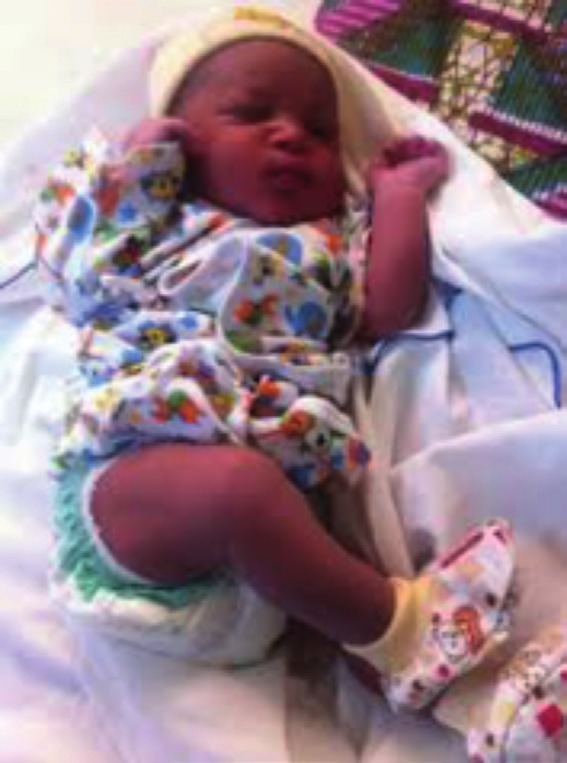
On August 7, 2017, the first TWE baby was born in Accra.

The Walking Egg npo emphasizes the importance of preventing infertility as part of integrated reproductive health programs and the need to improve the quality of (low tech) infertility care in the public health sector by means of standardized guidelines, training of health staff and improved counselling (Ombelet et al., 2009; Gerrits and Shaw, 2010; Gerrits, 2016).

As evidence-based, affordable solutions begin to drive global guidance within both public and private health care systems, access to care for the infertile couple will become one of the largest emerging fields in global medicine. We hope and believe that infertility care will be one of the most predominant components of future reproductive health care practice in Africa.

## Conclusion

The magnitude of childlessness in African countries has dimensions beyond its prevalence. Although reproductive health education and prevention of infertility are number one priorities, the need for accessible diagnostic procedures and new reproductive technologies is very high. Utilization of IVF is obviously associated with affordable access to ART which is related to insurance or public funding. In sub-Saharan Africa the success and sustainability of ART will depend, to a large extend, on our ability to optimise these techniques in terms of availability, affordability and effectiveness.

Accessible infertility treatment can only be successfully introduced if socio-cultural and economic prerequisites are fulfilled and governments can be persuaded to support their introduction. It is our duty to liaise with the relevant authorities to discuss the strengthening of infertility services, at the core of which lies the integration of infertility, contraceptive and maternal health services within public health care structures.

Registration of data and quality control programs are extremely important if African countries want to become a valuable player in the world of assisted reproduction. After a fascinating period of 40 years of IVF, only a small part of the African population benefits from these new technologies. Time has come to give equitable access to effective and safe infertility care in resource-poor African countries as well.

“In a world that needs vigorous control of population growth, concerns about infertility may seem odd, but the adoption of a small family norm makes the issue of involuntary infertility more pressing. If couples are urged to postpone or widely space pregnancies, it is imperative that they should be helped to achieve pregnancy when they so decide, in the more limited time they will have available”.

Mahmoud Fathalla, Former Director UNDP/UNFPA/UNICEF/WHO/World Bank Special Programme of Research, Development and Research Training in Human Reproduction
